# Clinical and Economic Evaluation of Acupuncture for Opioid-Dependent Patients Receiving Methadone Maintenance Treatment: The Integrative Clinical Trial and Evidence-Based Data

**DOI:** 10.3389/fpubh.2021.689753

**Published:** 2021-08-16

**Authors:** Hao Wen, Xiaojing Wei, Shuqi Ge, Jingchun Zeng, Wen Luo, Rouhao Chen, Yu Dong, Songhua Xiao, Yunfeng Lai, Liming Lu

**Affiliations:** ^1^Department of Neurology, The Sun Yat-sen Memorial Hospital of Sun Yat-sen University, Guangzhou, China; ^2^South China Research Center for Acupuncture and Moxibustion, Medical College of Acu-Moxi and Rehabilitation, Guangzhou University of Chinese Medicine, Guangzhou, China; ^3^First Affiliated Hospital of Guangzhou University of Chinese Medicine, Guangzhou, China; ^4^School of Medical Information Engineering, Guangzhou University of Chinese Medicine, Guangzhou, China; ^5^State Key Laboratory of Quality Research in Chinese Medicine, Institute of Chinese Medical Sciences, University of Macau, Taipa, China; ^6^South China Research Center for Acupuncture and Moxibustion, Medical College of Acu-Moxi and Rehabilitation, Guangzhou University of Chinese Medicine, Guangzhou, China; ^7^Evidence-Based Medicine and Data Science Centre, Guangzhou University of Chinese Medicine, Guangzhou, China

**Keywords:** opioid dependence, acupuncture - therapy, methadone maintenance therapy, clinical evaluation, economic evaluation, randomized controlled trial

## Abstract

**Objective:** From the health care and societal perspectives, this study aimed to evaluate the clinical and economic effects of acupuncture as an adjunctive therapy for patients receiving methadone maintenance treatment (MMT).

**Methods:** We conducted a parallel-arm RCT in China in 2019. Patients were included who met the diagnostic criteria and receive MMT for more than 30 days. Patients were randomly assigned to the exposed group (acupuncture plus MMT) or control group (MMT) at a 1:1 ratio. Daily methadone dosage, drug cravings using the VAS score, and insomnia using the Pittsburgh Sleep Quality Index (PSQI) were chosen as the effectiveness indexes, and the quality-adjusted life years (QALYs) was chosen as the utility index.

**Results:** Overall, 123 patients were included. The exposed group was significantly (*P* < 0.05) better than the control group in the improvement of daily methadone dosage (17.68 vs. 1.07), VAS (38.27 vs. 2.64), and PSQI (2.18 vs. 0.30). The QALY was 0.0784 (95%CI: 0.0761–0.0808) for the exposed group and 0.0762 (95%CI: 0.0738–0.0787) for the control group. The total cost of the exposed group (2869.50 CNY) was higher than the control group (2186.04 CNY). The ICER of daily methadone dosage (41.15), VAS (17.86), and PSQI (313.51) were shown to be economically efficient. While ICUR (310,663.64 CNY/QYLY) was higher than the cost suggested by WHO.

**Conclusion:** Acupuncture as an adjuvant therapy for MMT patients realizes its cost-effectiveness by reducing the dosage of methadone, improving drug cravings, and alleviating insomnia. It helps to improve quality of life, but since its cost exceeds what society is willing to pay, further study is needed.

## Introduction

Opioid use disorder (OUD) is a type of addictive disease that includes compulsive opioid use, increased tolerance to opioids, and obvious withdrawal reactions after stopping drug taking (1, 2). Evidence from The World Drug Report 2018 shows that approximately 31 million people worldwide suffer from OUD, which has caused the huge burden of serious disease and drug-related deaths (3). In the United States, approximately $1.02 trillion was spent on opioid use disorders and fatal opioid overdoses in 2017 (4). Incidence of fatal opioid overdose continue to surge in the United States (5). In China, about 1.8 million people use illicit drugs, of which 734,000 are opioid abusers, accounting for 40.8% of it (6). Opioid dependence have caused a heavy economic burden on patients and the health care systems worldwide.

Methadone maintenance treatment (MMT), an effective intervention for the treatment of opioid dependence, is by far the most widely used treatment (7, 8). MMT uses less dependency-inducing methadone to replace opioids, aiming to reduce opioid use through the principle of cross-tolerance. It is one of the current worldwide treatments for opioid dependents, reducing a range of social and public health problems caused by opioid abuse (9).

Though the widespread use of methadone in maintenance treatment for opioid dependence in many countries, MMT patients are often troubled by side effects, such as constipation, drowsiness, menstruation, decreased libido, and gastrointestinal symptoms (10). Besides, Lai's (11) study showed that there was still a high proportion, more than 30%, of patients who were caught re-taking drugs during MMT treatment. Despite this, it remains to be further studied whether the cause is drug addiction or methadone underdosing.

Acupuncture has been treating various diseases for thousands of years in China. As an alternative and complementary therapy, it is becoming increasingly popular in Western countries (12). Acupuncture has been recognized as an appropriate non-pharmacological therapy for the treatment of substance dependence by the World Health Organization (13). The existing studies have shown evidence that acupuncture might potentially reduce relapse through inhibiting attention bias to heroin (14), lowering the complications of drug dependence (15), and modifying morphine withdrawal syndrome (16). Compared with Western medicine, acupuncture has fewer side effects and limited adverse reactions. Several studies have found that acupuncture has potential effects in improving opioid dependence (17, 18). In the RCT of Chan 2014 (19), 60 patients treated with MMT were randomized to receive either true or sham acupuncture for a period of 4 weeks. Significant reductions in daily methadone consumption and improvements in sleep latency were observed in the true acupuncture group compared with the sham acupuncture group. A pilot RCT by Deng et al. (20) found that acupuncture helped reduce drug dose in patients who had already taken opioids and alleviate opioid cravings in patients who had not taken opioids. Another study by Crawford et al. (21) found that among 172 patients who had received at least four acupuncture treatments at the US Air Force Medical Center, the total prescription of morphine equivalent was reduced by 45%. A systematic review article by Baker et al. (22) concluded that auricular acupuncture may be effective as an adjuvant therapy to increase treatment retention and decrease methadone maintenance dosage. These studies proved the effectiveness of acupuncture for reducing drug use in different disease areas. Some have even suggested that it is better at pain relief than opioids (23–25). In addition, a randomized controlled trial we carried out proved that the effect of acupuncture as an adjunctive therapy of treating opioid dependence is better than methadone treatment alone (26).

Previous studies found that there is evidence that acupuncture improves opioid dependence. Whether this translates into better health outcomes for patients and fewer costs for the health care system needs to be investigated. And there is little known about the clinical and economic evaluations on acupuncture for opioid dependence patients, thus, this study aimed to conduct clinical and economic evaluation of acupuncture as an adjunctive therapy for patients receiving MMT in a randomized controlled trial. It is expected that the findings can provide references for clinicians to optimize treatment options.

## Methods

### Study Design

We implemented a randomized controlled trial (RCT) in which 135 patients were randomly assigned to a routine group (control group) or an acupuncture plus routine group (exposed group) *via* a central randomization system (SAS 9.4) in a 1:1 ratio. After completing the clinical trial, we conducted the cost-effectiveness analysis (CEA) and cost-utility analysis (CUA) on the collected data from the societal perspective. The protocol approved by the Ethics Committee of the First affiliated Hospital of Guangzhou University of Chinese Medicine (No. Y-2019-241) and registered in the Chinese Clinical Trial Registry (ChiCTR1900026357) has been published.

### Participants

Participants were recruited with OUD from the methadone clinic of the Substance Dependence Department of Guangzhou Huiai Hospital from October 2019 to September 2020. OUD was diagnosed according to the Diagnostic and Statistical Manual of Mental Disorders [Fifth Edition (DSM-V)] of the American Psychiatric Association. The specific inclusion and exclusion criteria are presented in Table 1.

**Table 1:** The inclusion and exclusion criteria of subjects.

**Inclusion criteria**	**Exclusion criteria**
(1) Male or female. (2) 18–60 years old. (3) Had received MMT for not <30 days. (4) Were able to sign the informed consent. (5) Had not received any kind of acupuncture therapy during the previous 3 months.	(1) Serious heart, liver, lung, or kidney disease. (2) Venereal disease or AIDS. (3) The presence of severe digestive disease and athrepsia. (4) Major psychosis. (5) The receipt of other treatment that may affect the efficacy evaluation of the present intervention. (6) An infection, inflammation, scar, or injury close to the site of the selected acupoints. (7) Pregnant or planning to become pregnant.

### Intervention

#### Exposed Group: Acupuncture Plus MMT

Participants received acupuncture plus MMT treatment in this group. Acupuncture was performed by a licensed acupuncturist. The selection of acupoint groups based on Jin's three-needle theory have specific names and contain three or four points. And three acupoints groups named Dingshen-zhen, Sishen-zhen, and Shouzhi-zhen were chosen in this study. The acupoints are described in detail in Appendix 1. Disposable stainless steel needles (Huatuo, Suzhou, China. Lengths and diameters 0.3 × 25 mm or 0.3 × 40 mm) were used in this group. Insertion was followed by manual stimulation, aiming to make patients feel the typical acupuncture sensation of de qi (considered to indicate effective needling, it is a sensation of soreness, numbness, distention, or radiating). Then the needles remained on the point for 30 min. Patients received a total of 18 sessions over a period of 6 weeks (3 times per week).

#### Control Group: MMT

Participants received MMT only. The dosage and reduction of methadone maintenance treatment are based on relevant national regulations (the details of the methadone instructions and reduction methods are in Appendix 2).

### Clinical Outcome Measures

#### Effectiveness Indexes

##### Daily Dosage of Methadone

The daily consumption of methadone was recorded from baseline (0 week) to the 2nd, 4th, and 6th weeks. One primary outcome measure was the change in the daily consumption of methadone from baseline to the 6th week. Enrolled patients went to the clinic to take methadone every day, and the dosage was strictly administered by the doctor which was recorded in the clinic's computer system.

##### VAS

The severity of the opioid craving was assessed using a 100-mm visual analog scale (VAS) at baseline (0 week) and the 2nd, 4th, and 6th weeks. The amount of opioid craving that a participant felt ranged across a continuum from 0 (no craving) to 100 (strong craving). The opioid craving VAS score was determined by measuring in millimeters from the left-hand end of the line to the point that the participant marked (27). The other primary outcome measure was the change in VAS from baseline to the 6th week.

##### PSQI

Sleep quality was assessed using the Chinese version of the Pittsburgh Sleep Quality Index (PSQI) (28) at baseline and at the 6th week. The Chinese version of the PSQI has demonstrated reliability and validity similar to that of the original language version (29). The PSQI evaluates sleep disturbances through subjective sleep quality, sleep duration, sleep latency, habitual sleep efficiency, sleep disturbances, daytime dysfunction, and the use of sleeping medication. Each item is graded on a 4-point scale (0 points to 3 points), which is summed for a total score (0 points to 21 points). Higher scores show that patients suffer a higher severity of sleep disturbance, and a total score higher than 5 indicates “poor sleep.”

#### Utility Index

The Quality adjusted life years (QALY) assessment was used as the utility index. The quality of life was assessed through the Chinese version of the SF-36 (30) at baseline and at the 6th week. The Chinese version of the SF-36 has demonstrated validity similar to that of the original language version (31). The quality of life was estimated by the SF-36 in eight domains: general health (GH), physical functioning (PF), role limitations due to physical problems (RP), role limitations due to emotional problems (RE), bodily pain (BP), social functioning (SF), vitality (VT), and general mental health (MH) (32). PF, RP, RE, BP, and SF assess the absence of limitations or disability, while GH, VT, and MH assess the positive state of well-being. Mid-range scores indicate no reported limitations or disabilities (33). In this study, higher scores indicate a better health status.

QALY was used to calculate the health utility value according to the corresponding coefficients of each dimension level in the SF-6D UK utility scoring model (health utility value = C + PF + RL + SF + PAIN + MH + VIT). Using the Area Under the Curve (AUC) method to calculate QALY (34–36), QALY is calculated as follows:

$$\begin{array}{l} QALY=\frac{1}{2}\overset{n-1}{\underset{i=0}{\sum }}\left(t_{i-1}-t_{i}\right)\left(y_{i}+y_{i-1}\right) \end{array}$$

In the above formula, “*y*_*i*_” represents the utility value of the health status of a survey object at time “*t*_*i*_” (*i* = 0, 1, 2……, *n*), and “*n*” represents the number of measurements taken by the survey object. This study is adjusted to be based on a time frame of 1 year.

Moreover, the subjects will be randomly asked to undergo a urine test to detect relapse of opioids.

### Selection and Determination of Cost

All costs were calculated with CNY as the unit. Expense information was collected through questionnaires, which were filled out by the patient or accompanying family members based on their actual expenses.

#### Direct Costs

Direct costs consist of direct medical costs and direct non-medical costs. In this study, direct medical costs include the costs for acupuncture treatment, physical examination, and urine tests. Direct non-medical costs are related to transportation expenses incurred by the trial.

The standard of acupuncture treatment fee comes from the “Price Summary List of Basic Medical Services in Guangdong Province” published by the Guangzhou Municipal Development and Reform Commission in December 2018, the price of acupuncture treatment is 33 CNY per session. Since methadone is a controlled drug in China and there is no reference price, we investigated the material dependence clinic of methadone at Huiai Hospital in Guangzhou. The results of the investigation are as follows: Methadone is provided by China Pharmaceutical Group (Shenzhen) Pingshan Pharmaceutical Co., Ltd. The dosage of MMT for patients is determined by doctors and adjusted reasonably according to the patient's requirements. Regardless of the dosage, patients only need to pay 10 CNY per time.

#### Indirect Costs

Indirect costs were considered as a result of lost productivity caused by a reduction in the effective working hours or the ability to work of the patient and his/her family due to morbidity. The unit labor value was based on the per capita GDP of Guangzhou in 2019. The indirect costs were measured as follows:

$$\begin{array}{l} \text{Indirect costs}=\text{Labor loss time}\times \text{Unit time labor value}\\ \left(\text{Unit time labor value}=\text{GDP per capita }÷\;365\text{ days }÷8\text{ h}\right). \end{array}$$

### Statistical Analysis

In this study, descriptive statistic analysis was conducted on the description of basic characteristics, comparative analysis of outcome, and cost. Measurement data were described by mean, and enumeration data were described by frequency and rate. CEA and CUA were conducted in the study. Further, incremental cost-effectiveness ratio (ICER) and incremental cost-utility ratio (ICUR) were adopted to determine which group would bring more economic benefit to patients.

In addition, only when ICER or ICUR is within the threshold standard range will it be applied. This study was classified according to the WHO's threshold level and China Guidelines for Pharmacoeconomic Evaluations (2020), taking 3-time per capita GDP as the threshold. According to China's per capita gross national product (GDP: 72,447 CNY) announced by the National Bureau of Statistics in 2020, the threshold for this study was determined to be 217,341 CNY.

Statistical analyses were conducted using SAS version 9.4 (SAS Institute Inc., Cary, NC), including SAS PROC MIXED for linear mixed models. Two-sided *P* < 0.05 indicated statistical significance.

### Sensitivity Analysis

Probabilistic sensitivity analysis was used to test the stability of the results. Probability sensitivity analysis was performed by the @RISK software, and its results are presented by the cost-effectiveness acceptability curves (CEAC).

## Results

**Figure 1:**
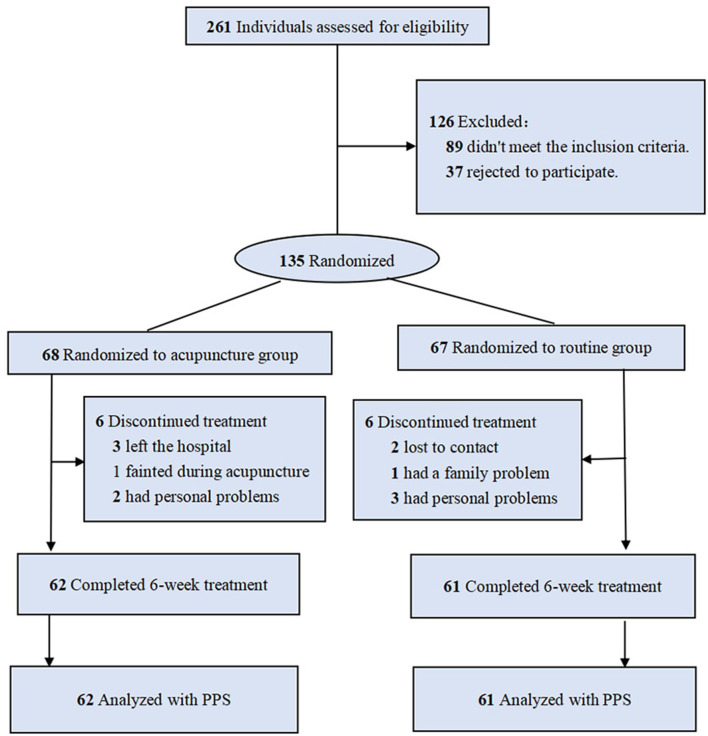
Flow chart.

From October 2019 to September 2020, 135 of the 326 patients screened were included at baseline and randomly assigned to the exposed group (*n* = 68) or the control group (*n* = 67). A total of 12 patients dropped out during the study period: 3 patients left the hospital, 1 patient fainted during acupuncture, 2 patients lost contact, and 6 patients had personal problems. Thus, we analyzed 123 patients who completed all treatments and after 6 weeks of follow-up. The flowchart of sampling and grouping is summarized in Figure 1.

### Baseline Characteristics

Patient baseline characteristics are listed in Table 2. There was little difference in age, sex, weight, marital status, job, education, and other personal conditions between the two groups. In addition, there was no significant difference in specific situation of drug use, such as years of opioid use, route of previous opioid use, daily dosage of opioid used before MMT, and positive rate of urine test. And except the sex proportion, all scores of standardized differences were <0.2. According to standardized difference scores, most of the baseline variables were balanced between the two groups.

**Table 2:** Characteristics at baseline (PPS).

**Variable**	**Exposed group**	**Control group**	**Standardized difference**
	**(*n* = 62)**	**(*n* = 61)**	
Age, mean (SD), y	51.79 (4.77)	51.08 (6.71)	0.02
Sex (male/female)	49/13	56/5	0.22
BMI, mean (SD), kg/m^2^	21.76 (3.68)	22.54 (3.57)	0.06
Marital status (%)			
Married	20 (32.3)	21 (34.4)	0.03
Single	17 (27.4)	22 (36.1)	0.13
Divorced	25 (40.3)	18 (29.4)	0.16
Unemployed rate (%)	51 (82.3)	51 (83.6)	0.02
Education (%)			
Primary school	11 (17.7)	18 (29.5)	0.18
Middle school	25 (40.3)	22 (36.1)	0.06
High school or university	26 (41.9)	21 (34.4)	0.11
Years of opioid use, mean (SD)	16.76 (6.12)	16.44 (8.54)	0.01
Route of previous opioid use (%)			
Injection	49 (79)	41 (67.2)	0.13
Nasal	13 (21)	18 (29.5)	0.18
Oral	0 (0)	2 (3.3)	0.09
Daily dosage of opioid used before MMT (g/d)	1.08 (1.15)	0.90 (1.14)	0.14
Urine test positive (%)	5 (8.1)	11 (18)	0.17

*PPS, per protocol set; SD, standard deviation; BMI, body mass index; MMT, methadone maintain treatment*.

*Standardized difference = difference in means or proportions divided by standard error. Imbalance was defined as an absolute value >0.20 (small effect size)*.

### Outcomes

#### Outcomes of Effectiveness

As shown in Table 3, the outcomes of both groups included daily dosage of methadone, VAS score, and PSQI score.

**Table 3:** Outcome comparison of the exposed group and control group (X ± SD).

**Outcome**	**Follow-up**	**Exposed group**	**Control group**	**Difference between the groups**	***P-*value**
Daily dosage of methadone (Unit: mg)	Week 0	52.44 ± 9.03	49.41 ± 17.05	3.066 ± 2.65	0.250
	Week 4	37.05 ± 6.23	48.54 ± 16.71	−11.71 ± 2.01	<0.001
	Week 6	34.76 ± 7.9	48.34 ± 17.44	−13.67 ± 2.11	<0.001
	**Effectiveness**	17.68 ± 1.50	1.07 ± 2.24	/	/
	*P-*value	<0.001	0.635	/	/
VAS score	Week 0	49.39 ± 22.78	46.30 ± 19.63	3.10 ± 4.05	0.447
	Week 4	26.69 ± 15.17	50.92 ± 13.16	24.23 ± 2.55	<0.001
	Week 6	11.39 ± 6.45	48.93 ± 13.03	37.54 ± 1.93	<0.001
	**Effectiveness**	38.27 ± 2.97	−2.64 ± 1.84	/	/
	*P-*value	<0.001	0.156	/	/
PSQI score	Week 0	10.35 ± 4.81	8.76 ± 4.95	1.58 ± 0.88	0.077
	Week 4	8.75 ± 4.65	8.73 ± 4.80	0.02 ± 0.82	0.985
	Week 6	8.18 ± 4.55	9.06 ± 5.00	0.93 ± 0.83	0.271
	**Effectiveness**	2.18 ± 0.52	−0.30 ± 0.42	/	/
	*P-*value	<0.001	0.477	/	/

*SD, standard deviation*.

*Repeated measures analysis of variance were used to analyze the individual effects of each factor due to interaction*.

##### Daily Dosage of Methadone

In the exposed group, the daily methadone dosage at the 6th week was reduced by an average of 17.68 mg compared to the 0 week and a *P* < 0.001 indicated a significant difference. While in the control group, the dosage was only reduced by 1.07 mg on average with no statistical significance (*P* = 0.595). Moreover, at the 6th week, it was statistically significant (*P* < 0.001) that the daily methadone dosage of the exposed group was 13.67 mg less than that of the control group.

##### VAS Score

In terms of VAS score, at the 6th week, the exposed group had a score of 11.39 ± 6.45 which compared to the baseline improved by 38.27 ± 2.97 (*P* < 0.001) after treatment, while the control group had a score of 48.93 ± 13.03 which had no improvement compared to the baseline (−2.64 ± 1.84, *P* = 0.156). Besides, the difference between the groups was significant at 37.54 ± 1.93 (*P* < 0.001).

##### PSQI Score

As to the PSQI score, the mean score of the exposed group was 8.18 ± 4.55 and the control group was 9.06 ± 5.00 at the 6th week. Compared with the baseline, the exposed group improved by 2.18 ± 0.52 (*P* < 0.001), while the control group did not improve (−0.30 ± 0.42, *P* = 0.477). And difference between the groups was 0.93 ± 0.83 with no significance.

#### Outcome of Utility

No significant difference was observed in health utility values between the exposed group and control group (*P* > 0.05), though health utility values of the exposed group showed an increasing trend over time (see Appendix 3).

The QALY score was 0.0784 (95%CI: 0.0761–0.0808) for the exposed group and 0.0762 (95%CI: 0.0738–0.0787) for the control group. And the exposed group had more 0.0022 QALYs than the control group.

### Costs

Details of the costs are shown in Table 4.

**Table 4:** Description of different types of costs (Unit: CNY).

**Cost type**	**Group**	**Mean**	***SD***	***P_**25**_***	***M***	***P_**75**_***	***P value***
**Total costs** ^**a**^	Exposed group	2,869.5	1,561.91	1,406	2,426	4,296.25	0.008
	Control group	2,186.04	1,247.36	899.5	2,422.5	2,844.5	—
**Total direct costs** ^**b**^	Exposed group	1,416.29	172.75	1,369.5	1,409.5	1,470	<0.001
	Control group	834.71	113.39	795.5	819.5	903.5	—
**Total direct medical costs** ^**c**^	Exposed group	1,303.94	98.72	1,317.5	1,337.5	1,341.5	<0.001
	Control group	729.66	64.03	723.5	733.5	747.5	—
Acupuncture fee	Exposed group	594	—	—	—	—	—
	Control group	—	—	—	—	—	—
Physical examination fee	Exposed group	286.44	98.72	300	320	324	0.192
	Control group	306.16	64.03	300	310	324	—
Urine test fee	Exposed group	3.5	—	—	—	—	—
	Control group	3.5	—	—	—	—	—
Medicine fee	Exposed group	420	—	—	—	—	—
	Control group	420	—	—	—	—	—
**Direct non-medical cost** ^**d**^	Exposed group	112.35	165.70	72	72	144	0.766
	Control group	105.05	96.38	72	72	162	—
**Indirect costs** ^**e**^	Exposed group	1,453.21	1,530.28	0	958.5	2,875.5	0.690
	Control group	1,351.33	1,278.52	0	1,917	1,917	—

*SD, standard deviation*.

a *Total costs are the sum of direct and indirect costs*.

b *Direct costs include direct medical expenses and direct non-medical expenses*.

c *Total direct medical costs include acupuncture fee, physical examination fee, urine examination fee, and drug fee*.

d *Direct non-medical cost is related to the transportation expenses incurred by the trial*.

e *Indirect cost is calculated by multiplying the labor lost time by 53.25 CNY per hour*.

#### Total Costs

The mean total costs were 2,869.50 CNY and 2,186.04 CNY, respectively for the exposed group and control group. Total cost in the exposed group was higher than the control group, and the difference of the mean total costs was statistically significant between the two groups (*P* < 0.001).

#### Direct Costs

In terms of direct medical costs, the mean was 1,303.94 CNY (exposed group) and 729.66 CNY (control group) with a *P* ≤ 0.05 indicating a significant difference. The mean of direct non-medical costs was 112.35 CNY (exposed group) vs. 105.05 CNY (control group), and the *P* = 0.766 indicated that the difference was not significant.

#### Indirect Costs

The mean of indirect costs was 1,453.21 CNY and 1,351.33 CNY with the median of 958.50 CNY and 1,917.00 CNY for the exposed group and control group, respectively.

In sum, both groups showed a significant difference in direct medical costs (*P* < 0.001) and a non-significant difference in direct non-medical costs (*P* = 0.766) or indirect costs (*P* = 0.690). Overall, all types of costs were higher in the exposed group than in the control group.

### Results of CEA

As shown in Table 5, regarding daily methadone dosage, the exposed group was associated with a more reduction of daily methadone dosage (16.61) compared with the control group, and with a higher cost (683.46 CNY). This means that increasing the effective rate by 1% cost an extra 41.15 CNY.

**Table 5:** Results of CEA.

	**Cost (CNY)**	**Effectiveness**	**CER**	**ΔC (CNY)**	**ΔE**	**ICER**
**Daily methadone dosage**						
Exposed group	2,869.50	17.68	162.30	683.46	16.61	41.15
Control group	2,186.04	1.07	2,043.03			
**VAS score**						
Exposed group	2,869.50	38.27	74.98	683.46	38.27	17.86
Control group	2,186.04	0	-			
**PSQI**						
Exposed group	2,869.50	2.18	1,316.28	683.46	2.18	313.51
Control group	2,186.04	0	-			

*CEA, cost-effectiveness analysis; CNY, China Yuan; CER, cost-effectiveness ratio; C, cost; E, effectiveness; ICER, incremental cost-effectiveness ratio*.

*ΔC = Cost_exposed Gourp_ – Cost_control group_; ΔE = Effectiveness_exposed Gourp_ – Effectiveness_control group_; ICER = ΔC/ΔE*.

Regarding VAS score, compared with the control group, the exposed group was associated with a higher cost (683.46 CNY) and a higher effective rate (38.27). This means that increasing the effective rate by 1% cost an extra 17.86 CNY.

In terms of PSQI score, compared with the control group, the exposed group was associated with a higher cost (683.46 CNY) and a higher effective rate (2.18). This means that increasing the effective rate by 1% cost an extra 313.51 CNY.

### Results of CUA

It was calculated that the incremental cost of the exposed group was 683.46 CNY with 0.0022 QALY as the incremental utility. The ICUR under the exposed group was 310,663.64 CNY/QALY, indicating that it may not be economical as an auxiliary treatment for patients in MMT given a willingness to pay 217,341 CNY per QALY (triple China's per capita GDP in 2020).

### Sensitivity Analysis

**Figure 2:**
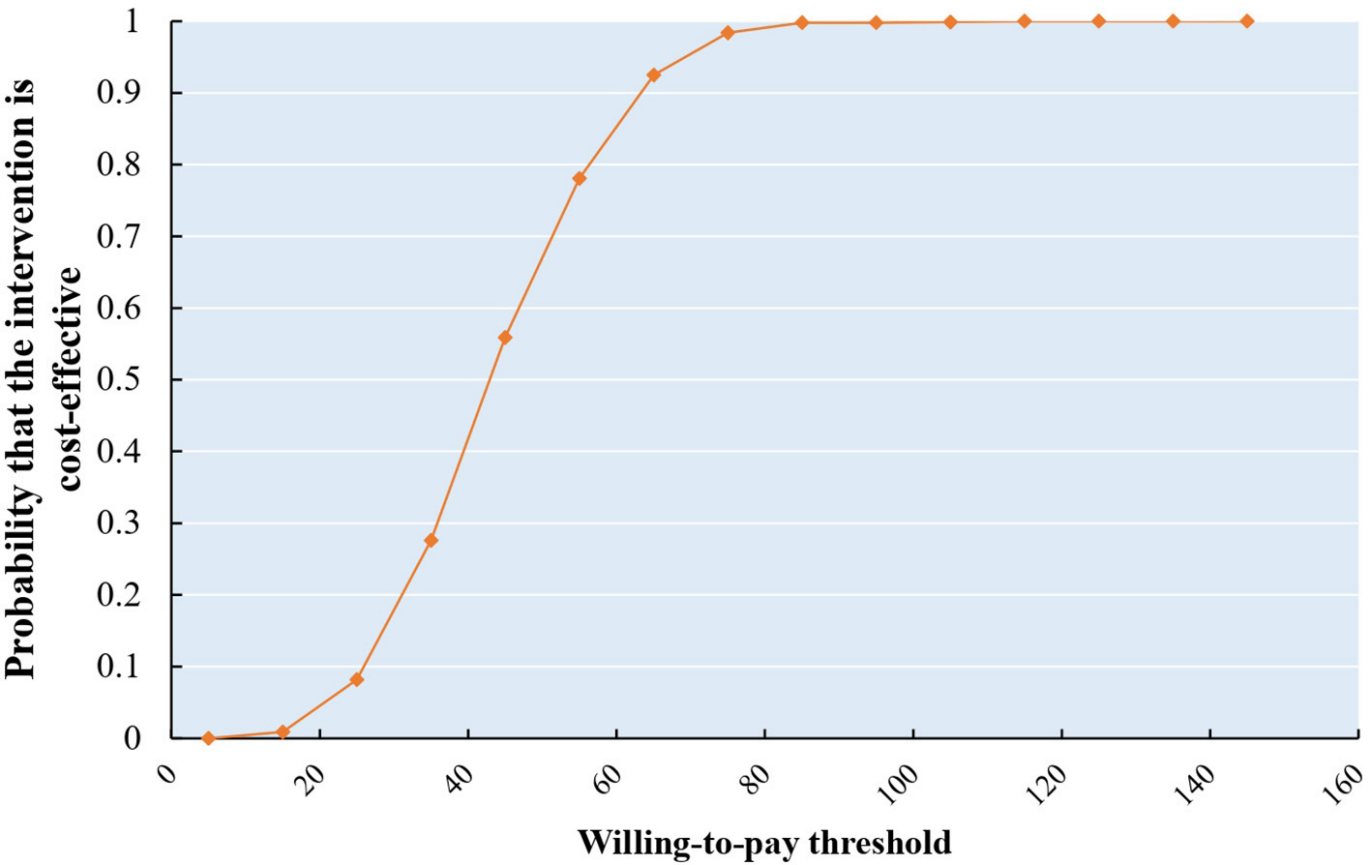
CEAS of reduction in daily methadone dosage.

**Figure 3:**
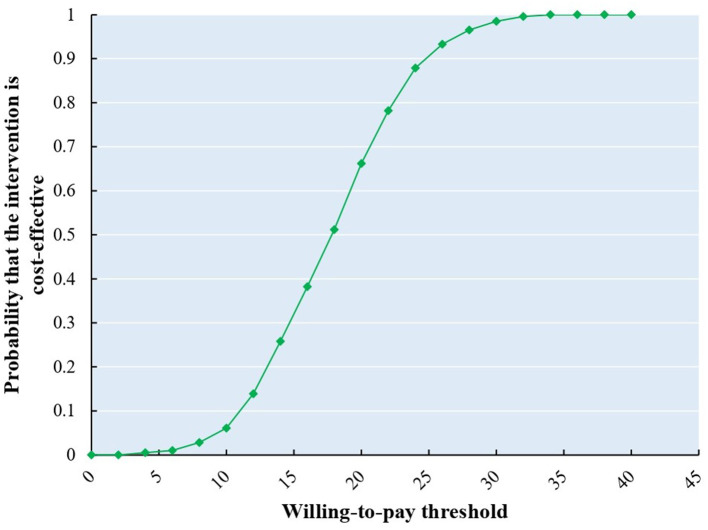
CEAS of improvement of opioid craving (VAS).

**Figure 4:**
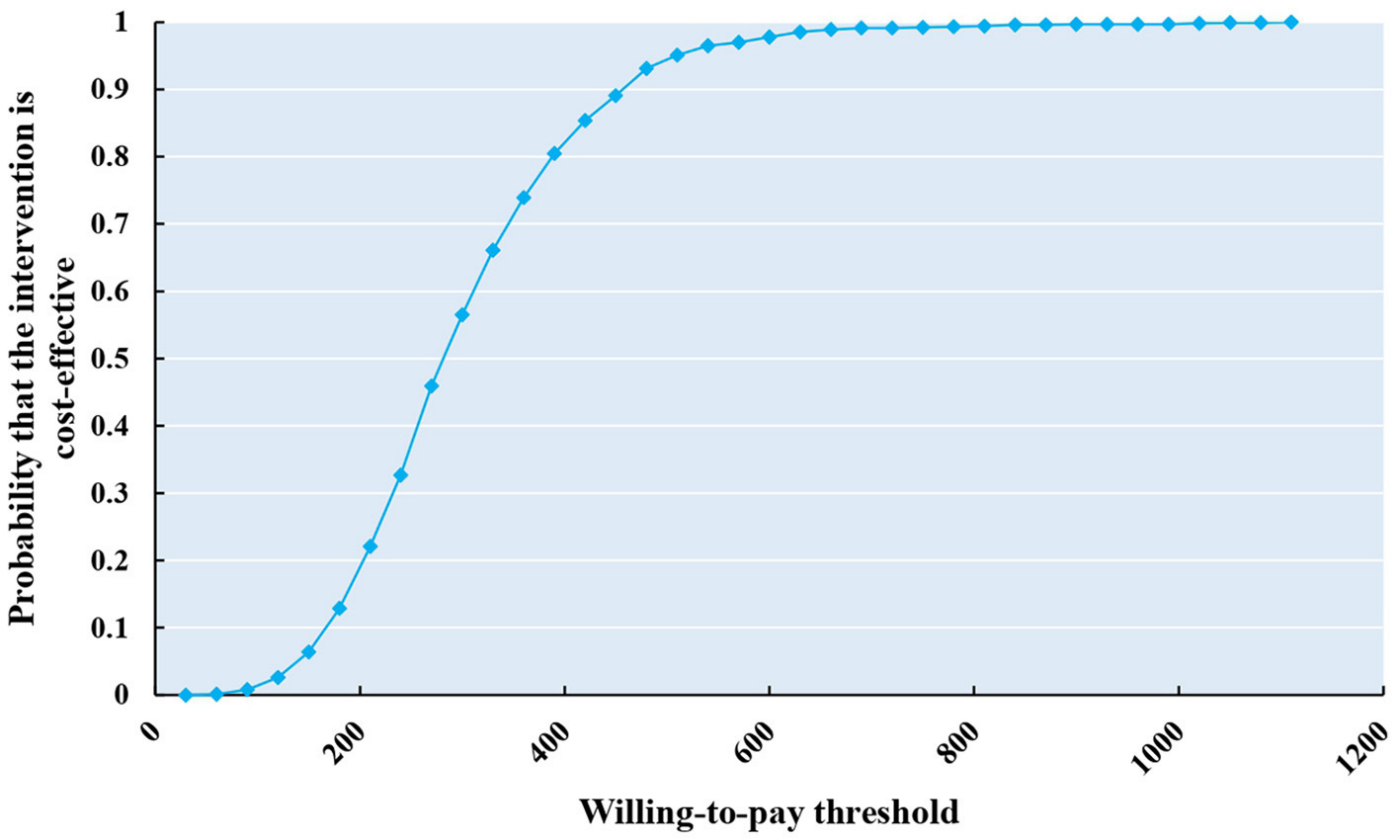
CEAS of improvement of insomnia (PSQI).

The results of probability analysis are shown in Figures 2–4. The CEAC represents how the likelihood of a treatment regimen having a cost-effectiveness advantage changes as the threshold changes. The CEAC shows that for the MMT patients, the addition of acupuncture in MMT to reduce methadone dosage is cost-effective, with a 50% probability of being cost-effective at a willingness to pay <50 CNY. At the same time, to improve the VAS and PSQI, the willingness to pay is <20 CNY and 400 CNY, respectively. When triple China's per capita GDP (217,341 CNY) indicates a cost-effective threshold, all three CEAC reach a probability of 100%.

## Discussion

In this study, we focused on the clinical and economic evaluation of acupuncture plus MMT treatment, comparing with MMT only, which has been proved to be clinically effective in the treatment of opioid dependence. We compared the MMT with acupuncture plus MMT through clinical and economic evaluation outcome indicators including daily dosage of methadone, VAS score, PSQI score, and SF-36 score, to evaluate the treatment economy and effectiveness of acupuncture treatment in a randomized controlled trial.

By the end of 2020, China had about 1.80 million drug users (6). The United States has become the epicenter of the opioid epidemic and is experiencing an ongoing overdose crisis (4, 37). In 2017, the cost of opioid use disorders and fatal opioid overdoses was estimated at $1.02trillion (34). Methadone maintenance therapy is currently the most widely used and effective treatment for opioid dependence worldwide (38–40). However, higher relapse rates persist during treatment (41, 42). But methadone itself also has many disadvantages. Long-term dependence on maintenance agents can cause insomnia, constipation, decreased libido, decreased sexual power, sweating, and other symptoms (40, 43, 44). Moreover, methadone is a synthetic opioid, and patients treated with MMT may become physically dependent and may experience methadone withdrawal symptoms (45). Methadone overdose has become a growing phenomenon in some countries (45). It has even led to a number of deaths from methadone overdoses (46). Therefore, in the treatment of OUD patients, methadone dependence and reduction of methadone dosage should also be paid attention to.

As a non-pharmacological method, acupuncture therapy is safe, has no side effects, and is not addictive. Its therapeutic effect on addictive diseases has been confirmed in animal and human studies (17, 47), and in addition, the therapeutic effects of acupuncture are also shown in various categories of diseases. A study of Eccleston et al. (48) showed that patients with chronic non-cancer pain after 6 weeks of acupuncture had lower consumption of opioids compared with the placebo group. Acupuncture may be an effective and safe pain-reducing treatment in palliative care for cancer patients that can help reduce the side effects of opioid use, including insomnia, constipation, and anxiety, according to a systematic review published in the BMJ (49). Chen et al. (50) conducted a meta-analysis which analyzed nine articles from the US, UK, and China involving 1,063 patients and concluded that acupuncture could be an effective treatment for OUD, among them, electroacupuncture could relieve opioid cravings and depression symptoms, and transcutaneous acupoint electrical stimulation is beneficial to the improvement of insomnia and anxiety. Following this, our research complements the effectiveness of manual acupuncture for OUD. Compared with methadone maintenance alone, the addition of acupuncture therapy could not only reduce the consumption of methadone, but also lowered cravings and improved insomnia. The therapeutic effect was better than methadone maintenance therapy alone (44, 51). These results are consistent with the results of our study. Our study outcome directly shows that in the group that was treated with acupuncture therapy, the daily dosage of methadone was significantly decreased, drug cravings based on the VAS score table were greatly lessened, and the insomnia score was also improved. Acupuncture therapy is also effective for other types of addictive diseases. Zeng et al. (52) observed that electroacupuncture helped improve psychiatric symptoms and anxiety and depression in methamphetamine addicts during abstinence in a 68-sample RCT using placebo needles as the control group. In a three-arm trial conducted at a German addiction center, Krause et al. (53) observed that ear acupuncture may improve autonomic cardiac function in alcohol addicts. We can say that acupuncture therapy as adjuvant treatment for addictive diseases has considerable potential and development prospects.

Furthermore, it should be noted that acupuncture treatment is cost-effective. In this study, after 6 weeks of treatment, we see that the CER value of reducing daily methadone dosage in the group that had been treated with acupuncture was dramatically lower than the MMT only group. In addition, as to the ICER, although the incremental cost-effectiveness ratio of acupuncture was not high, acupuncture's health value from the perspective of effectiveness deserves serious consideration, such as reduced drug usage, reduced drug cravings, and improvement of sleep. In other words, the extra cost it pays to get better results is in line with economic values. From the perspective of effectiveness, it is proven that acupuncture therapy has cost-effectiveness. But the cost-utility of acupuncture is not obvious. Its advantages are not positive either from a CUR or ICUR perspective. This may be due to the fact that acupuncture therapy mainly improves opioid dependence symptoms, while QALY is a comprehensive indicator, including physical, psychological, and social aspects. Therefore, its effect on QALY improvement is not obvious. In all, these results suggest that the short-term economic benefits of acupuncture treatment in MMT patients are certain, and the long-term benefits can be further explored.

This research is meaningful. OUD imposes a significant economic burden on both individuals and society. It is of great significance to learn what method we choose to achieve better benefits with fewer costs to reduce the economic burden brought by OUD. We conducted this economic evaluation on acupuncture as an adjuvant therapy for MMT patients from a social perspective. The purpose was to provide decision-makers with an economic and effective reference for treatment strategies. Given that economic benefits of acupuncture are identified above, choosing acupuncture as an adjuvant treatment for MMT patients has the benefits of low expense, reliable effects, and security.

The strengths of this study are obvious. First, the pragmatic study design allowed the evaluation of clinical data of acupuncture for opioid-dependent patients receiving MMT in the RCT. Second, we collected both effectiveness indexes (daily dosage of methadone, VAS, and PSQI) and a utility index (SF-36) to evaluate clinical outcomes, whereas most evaluation studies only collected effectiveness indexes. Third, different types of costs were included in the evaluation. Fourth, it provides a new alternative treatment for opioid-dependent patients.

However, this study has some limitations which can be addressed in future studies. Firstly, the study had a relatively small sample size which may introduce uncertainty in the clinical results. Secondly, there was a gender difference between the two groups due to the absence of some participants. Though there is no evidence that gender difference would affect the efficacy of acupuncture on addictive diseases so far, it is an undeniable pity for us. And we may investigate this further through real-world studies. Thirdly, in the analysis of QALY, it is possible that a substantial QALY difference cannot be detected due to the short follow-up time. Until our study was conducted, no Chinese value set based on the mainland Chinese population was available and we used the UK population-based value set for the calculation of QALYs. So it is not clear whether our utility result is acceptable. We have planned to launch a new clinical trial next, which will have a larger sample size and a longer period of treatment and follow-up and use the Chinese-specific value set for the calculation of QALYs. Last, our country lacks authoritative data for reference, which leads to controversy over socially acceptable cost to raise a QALY cost. And taking foreign social willingness to pay standards as a reference may lead to misjudgment of the results (34). Hence, research on the treatment of MMT patients with acupuncture therapy still needs further development and improvement.

## Conclusion

Acupuncture therapy showed advantages in reducing the daily dosage of methadone and improving VAS score and PSQI score for opioid-dependent patients receiving MMT when compared with routine treatment. The results of this study also showed the dominating cost-effectiveness advantage and potential cost-utility advantage for the exposed group. Therefore, this study can help to inform prescription practice for opioid-dependent patients receiving MMT, and the health care system can adjust or formulate policies to reduce the disease burden of opioid-dependent patients receiving MMT and improve their quality of life.

## Data Availability Statement

The original contributions presented in the study are included in the article/Supplementary Material, further inquiries can be directed to the corresponding author/s.

## Ethics Statement

The studies involving human participants were reviewed and approved by The Ethics Committee of the First Affiliated Hospital of Guangzhou University of Chinese Medicine (No. Y-2019-241). The patients/participants provided their written informed consent to participate in this study.

## Author Contributions

LL, YL, and SX contributed to the conception, design of the study, and contributed to the critical revision of the article for important intellectual content. HW and XW drafted the manuscript. HW, XW, JZ, and SG performed the acupuncture treatment for patients. HW, XW, and YL analyzed data. WL, RC, and YD collected and registered data. All authors contributed to manuscript revision, read, and approved the submitted version.

## Conflict of Interest

The authors declare that the research was conducted in the absence of any commercial or financial relationships that could be construed as a potential conflict of interest.

## Publisher's Note

All claims expressed in this article are solely those of the authors and do not necessarily represent those of their affiliated organizations, or those of the publisher, the editors and the reviewers. Any product that may be evaluated in this article, or claim that may be made by its manufacturer, is not guaranteed or endorsed by the publisher.

## References

[1] Reimer J Vogelmann T Trümper D Scherbaum N. Opioid use disorder in Germany: healthcare costs of patients in opioid maintenance treatment. Subst Abuse Treat Prev Policy. (2019) 14:57. 10.1186/s13011-019-0247-9 31842942 PMC6916156

[2] Yang F Hao W. Application prospect of compound buprenorphine in treating opioid use disorders in China. Chin J Drug Depend. (2020) 29:169–75. 10.13936/j.cnki.cjdd1992.2020.03.002

[3] Nations U. Office of Drug and Crime: World Drug Report 2018. Vienna: United Nations publication, Sales No. *E18XI9*. (2018).

[4] Florence C Luo F Rice K. The economic burden of opioid use disorder and fatal opioid overdose in the United States, 2017. Drug Alcohol Depend. (2021) 218:108350. 10.1016/j.drugalcdep.2020.108350 33121867 PMC8091480

[5] Larochelle MR Wakeman SE Ameli O Chaisson CE McPheeters JT Crown WH. Relative cost differences of initial treatment strategies for newly diagnosed opioid use disorder: a cohort study. Med Care. (2020) 58:919–26. 10.1097/MLR.0000000000001394 32842044 PMC7641182

[6] Drug Situation Report of China in 2020 [N]. China Drug Control Report, 2021-07-23(003).

[7] Mattick RP Breen C Kimber J Davoli M. Methadone maintenance therapy versus no opioid replacement therapy for opioid dependence. Cochrane Database Syst Rev. (2009) 2009:CD002209. 10.1002/14651858.CD002209.pub2 12519570

[8] Somogyi AA Barratt DT Ali RL Coller JK. Pharmacogenomics of methadone maintenance treatment. Pharmacogenomics. (2014) 15:1007–27. 10.2217/pgs.14.56 24956254

[9] Li Y Zhao Y Wang B Liu C. The evaluation of quality of life and its influencing factors of opioid dependent patients with methadone maintenance treatment. Chin J Drug Depend. (2020) 29:218–23.

[10] Zhu C. Analysis of adverse conditions during methadone maintenance treatment for heroin-dependent persons. Electr J Clin Med Lit. (2019) 6:86. 10.16281/j.cnki.jocml.2019.35.057

[11] Lai W. The analysis of the causes of methadone maintenance therapy for loss in patients. Chin J Drug Depend. (2007) 04:299–301. 23444245

[12] Kim YS Jun H Chae Y Park HJ Kim BH Chang IM. The practice of Korean medicine: an overview of clinical trials in acupuncture. Evid Based Complement Alternat Med. (2005) 2:325–52. 10.1093/ecam/neh102 16136212 PMC1193543

[13] Culliton PD Kiresuk TJ. Overview of substance abuse acupuncture treatment research. J Altern Complement Med. (1996) 2:149–59, 161–5. 10.1089/acm.1996.2.149 9395651

[14] Jiang YP Liu H Xu P Wang Y Lu G-H. Effect of electro-acupuncture intervention on cognition attention bias in heroin addiction abstinence-a dot-probe-based event-related potential study. Chin J Integr Med. (2011) 17:267–71. 10.1007/s11655-011-0698-y 21509669

[15] Chen YH Ivanic B Chuang CM Lu DY Lin JG. Electroacupuncture reduces cocaine-induced seizures and mortality in mice. Evid Based Complement Alternat Med. (2013) 2013:134610. 10.1155/2013/134610 23690833 PMC3652148

[16] Wu JM Wei DY Luo YF Xiang XY. Clinic research on heroin de-addiction effects of acupuncture and its potentiality of preventing relapse. Zhong Xi Yi Jie He Xue Bao. (2003) 1:268–72. 10.3736/jcim20030412 15339529

[17] Fan AY Miller DW Bolash B Bauer M McDonald J Faggert S. Acupuncture's role in solving the opioid epidemic: evidence, cost-effectiveness, and care availability for acupuncture as a primary, non-pharmacologic method for pain relief and management-White Paper 2017. J Integr Med. (2017) 15:411–25 10.1016/S2095-4964(17)60378-9 29103410

[18] Wu MS Chen KH Chen IF Huang SK Tzeng PC Yeh ML. The efficacy of acupuncture in postoperative pain management: a systematic review and meta-analysis. PLoS ONE. (2016) 11:e0150367. 10.1371/journal.pone.0150367 26959661 PMC4784927

[19] Chan YY Lo WY Li TC Shen LJ Yang SN Chen YH Lin JG. Clinical efficacy of acupuncture as an adjunct to methadone treatment services for heroin addicts: a randomized controlled trial. Am J Chin Med. (2014) 42:569–86. 10.1142/S0192415X14500372 24871652

[20] Deng G Giralt S Chung DJ Landau H Siman J Li QS. Reduction of opioid use by acupuncture in patients undergoing hematopoietic stem cell transplantation: secondary analysis of a randomized, sham-controlled trial. Pain Med. (2020) 21:636–42. 10.1093/pm/pnz190 31498394 PMC7060400

[21] Crawford P Penzien DB Coeytaux R. Reduction in Pain medication prescriptions and self-reported outcomes associated with acupuncture in a military patient population. Med Acupunct. (2017) 29:229–31. 10.1089/acu.2017.1234 28874924 PMC5580367

[22] Baker TE Chang G. The use of auricular acupuncture in opioid use disorder: a systematic literature review. Am J Addict. (2016) 25:592–602. 10.1111/ajad.12453 28051842

[23] Lee JH Choi TY Lee MS Lee H Shin BC Lee H. Acupuncture for acute low back pain: a systematic review. Clin J Pain. (2013) 29:172–85. 10.1097/AJP.0b013e31824909f9 23269281

[24] Haake M Muller HH Schade-Brittinger C Basler HD Schäfer H Maier C. German Acupuncture Trials (GERAC) for chronic low back pain: randomized, multicenter, blinded, parallel-group trial with 3 groups. Arch Intern Med. (2007) 167:1892–8. 10.1001/Archinte.167.17.1892 17893311

[25] Lewis RA Williams NH Sutton AJ Burton K Din NU Matar HE. Comparative clinical effectiveness of management strategies for sciatica: systematic review and network meta-analyses. Spine J. (2015) 15:1461–77. 10.1016/j.spinee.2013.08.049 24412033

[26] Wen H Xu S Zeng J Ge S Liao Y Tang C. Effect of acupuncture for methadone maintenance treatment patients: study protocol of a randomized clinical trial. Trials. (2020) 21:1003. 10.1186/s13063-020-04930-x 33287868 PMC7720473

[27] Wewers ME Lowe NKA. critical review of visual analogue scales in the measurement of clinical phenomena. Res Nurs Health. (1990) 13:227–36. 10.1002/nur.4770130405 2197679

[28] Buysse DJ Reynolds CF Monk TH Berman SR Kupfer DJ. The Pittsburgh sleep quality index: a new instrument for psychiatric practice and research. Psychiatry Res. (1989) 28:193–213. 10.1016/0165-1781(89)90047-4 2748771

[29] Tsai P-S Wang S-Y Wang M-Y Su CT Yang TT Huang CJ. Psychometric evaluation of the Chinese version of the Pittsburgh sleep quality index (CPSQI) in primary insomnia and control subjects. Qual Life Res. (2005) 14:1943–52. 10.1007/s11136-005-4346-x 16155782

[30] Li Lu Wang Hongmei SHEN Yi. Development and psychometric tests of a Chinese version of the SF-36 health survey scales. Chin J Preven Med. (2002) 02:38–42. 12410965

[31] Tseng H-M. Lu J-fR, Gandek B. Cultural issues in using the SF-36 health survey in Asia: results from Taiwan. Health Qual Life Outcomes. (2003) 1:72. 10.1186/1477-7525-1-72 14641915 PMC385291

[32] Ware JE Sherbourne CD. The MOS 36-item short-form health survey (SF-36). I Conceptual framework and item selection. Med Care. (1992) 30:473–83. 10.1097/00005650-199206000-00002 1593914

[33] McHorney CA Ware JE Raczek AE. The MOS 36-item short-form health survey (SF-36): II. Psychometric and clinical tests of validity in measuring physical and mental health constructs. Med Care. (1993) 31:247–63. 10.1097/00005650-199303000-00006 8450681

[34] Wu J. Cost-utility analysis series 4 - definition and calculation of quality adjusted life years (QALYs). China J Pharm Econ. (2008) 04:30–5.

[35] Han S Ye L. Review and introduction of quality adjusted life years. China J Pharm Econ. (2012) 06:12–5.

[36] Liu X Li S Chen G. Development of the short form health survey and introduction of short form 6-dimention(SF-6D). Chin Health Econ. (2019) 38:8–11.

[37] Gong C-Z Liu W. Acupuncture and the opioid epidemic in America. Chin J Integ Med. (2018) 24:323–7. 10.1007/s11655-018-2989-z 29752610

[38] Cheng Z Chen G Dai M Luo W Lyu P Cao XB. New psychoactive substances abuse among patients with access to methadone maintenance treatment in Jiangsu province: a case-control study. Chin J Epidemiol. (2018) 39:625–30. 10.3760/cma.j.issn.0254-6450.2018.05.016 29860806

[39] Salsitz E Wiegand T. Pharmacotherapy of opioid addiction: “putting a real face on a false demon”. J Med Toxicol. (2016) 12:58–63. 10.1007/s13181-015-0517-5 26567033 PMC4781801

[40] Yee A Danaee M Loh HS Sulaiman AH Ng CG. Sexual dysfunction in heroin dependents: a comparison between methadone and buprenorphine maintenance treatment. PLoS ONE. (2016) 11:e0147852. 10.1371/journal.pone.0147852 26820154 PMC4731474

[41] Department of Health. Task Force to Review Services for Drug Misusers: Report of an Independent Review of Drug Treatment Services in England. London: HMSO (1996).

[42] Department of Health. Drug Misuse and Dependence: Guidelines on Clinical Management. London: HMSO (1999).

[43] Dole VP Nyswander ME Kreek MJ. Narcotic blockade. J Psychoactive Drugs. (1991) 23:232. 1765882

[44] Wu SLY Leung AWN Yew DTW. Acupuncture for detoxification in treatment of opioid addiction. East Asian Arch Psychiatry. (2016) 26:70–6. 27377488

[45] Reingardiene D Jodziuniene L Lazauskas R. Metadonas ir gydymo juo pavojai [Methadone treatment and its dangers]. Medicina (Kaunas). (2009) 45:419–25. Lithuanian. 10.3390/medicina45050053 19535889

[46] Tjagvad C Skurtveit S Linnet K Andersen LV Christoffersen DJ Clausen T. Methadone-related overdose deaths in a liberal opioid maintenance treatment programme. Eur Addict Res. (2016) 22:249–58. 10.1159/000446429 27246839

[47] Chen Z Wang Y Wang R Xie J Ren Y. Efficacy of acupuncture for treating opioid use disorder in adults: a systematic review and meta-analysis. Evidence-based Compl Altern Med. (2018) 2018:1–15. 10.1155/2018/3724708 30622598 PMC6304557

[48] Windmill J Fisher E Eccleston C Derry S Stannard C Knaggs R Moore RA. Interventions for the reduction of prescribed opioid use in chronic non-cancer pain. Cochrane Database Syst Rev. (2013) 9:CD010323. 10.1002/14651858.CD010323 23996347

[49] Yang J Wahner-Roedler DL Zhou X Johnson LA Do A Pachman DR. Acupuncture for palliative cancer pain management: systematic review. BMJ Support Palliat Care. (2021) bmjspcare-2020-002638. [Epub ahead of print]. 10.1136/bmjspcare-2020-002638 33441387 PMC8380897

[50] Chen Z Wang R Zhang M Wang Y Ren Y. Acupuncture combined with medication for opioid use disorder in adults: a protocol for systematic review and meta-analysis. BMJ Open. (2020) 10:e034554. 10.1136/bmjopen-2019-034554 32565455 PMC7310998

[51] Chen L. Clinical observation of 34 cases of heroin withdrawal symptoms treated by acupuncture and methadone. Jiangsu J Med Sci. (2005) 09:32–3.

[52] Zeng L Tao Y Hou W Zong L Yu L. Electro-acupuncture improves psychiatric symptoms, anxiety and depression in methamphetamine addicts during abstinence: a randomized controlled trial. Medicine (Baltimore). (2018) 97:e11905. 10.1097/MD.0000000000011905 30142795 PMC6112927

[53] Krause F Penzlin AI Ritschel G Barlinn K Reichmann H Weidner K. Randomized controlled three-arm study of NADA acupuncture for alcohol addiction. Addict Behav. (2020) 110:106488. 10.1016/j.addbeh.2020.106488 32599496

