# Supplementary figures and images for: Clinical and Economic Evaluation of Acupuncture for Opioid-Dependent Patients Receiving Methadone Maintenance Treatment: The Integrative Clinical Trial and Evidence-Based Data
Source: Front Public Health. 2021 Aug 16;9:689753. doi: 10.3389/fpubh.2021.689753 (PMC8415360; doi:10.3389/fpubh.2021.689753)

## Appendix 1: Locations of Acupuncture points used in the study

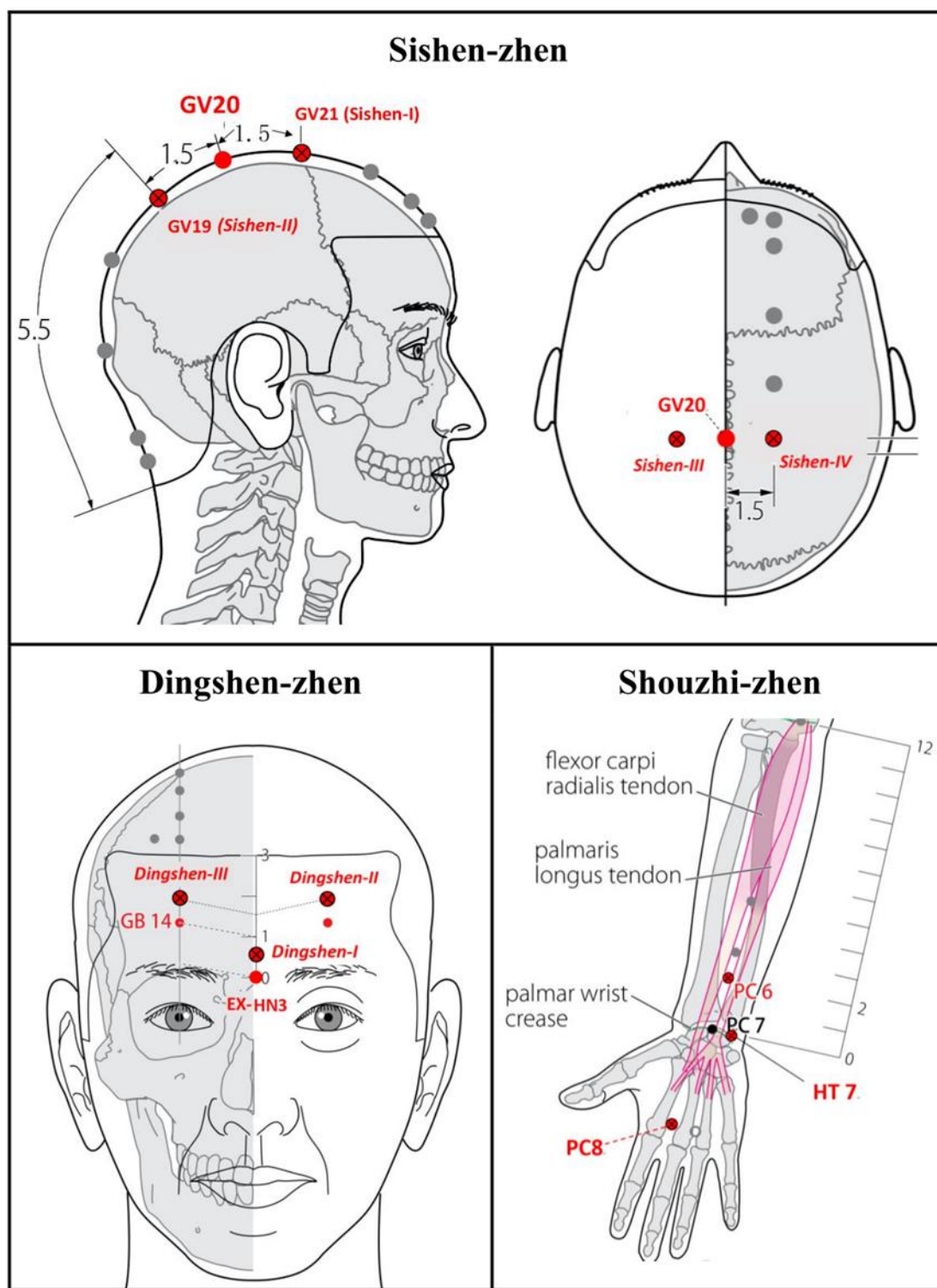

Supplement: Supplementary file 1 [file Data_Sheet_1.pdf]
