# Supplementary material for: Clinical and Economic Evaluation of Acupuncture for Opioid-Dependent Patients Receiving Methadone Maintenance Treatment: The Integrative Clinical Trial and Evidence-Based Data
Source: Front Public Health. 2021 Aug 16;9:689753. doi: 10.3389/fpubh.2021.689753 (PMC8415360; doi:10.3389/fpubh.2021.689753)
Supplement: Supplementary file 2 [file Table_1.docx]

**Appendix 2: Methadone dosage and instructions**

| Item | Instructions |
| --- | --- |
| 1. [Dose equivalent](C:/Users/meishaonv/AppData/Local/youdao/dict/Application/8.9.6.0/resultui/html/index.html#/javascript:;) | 1mg methadone=2mg heroin=4mg morphine=20mg methadine |
|  |  |
| 1. Determination of the first dose | ①Daily increasing method: use 20-40mg on the first day, and then increase by 5-10mg every 2 to 3 days until the patient's withdrawal symptoms can be completely controlled and the patient feels well. It usually takes 15 to 30 days to increase to the appropriate maintenance dose for the patient.  ② Twice a day method: According to conventional experience, the morning treatment dose is 20-30 mg, and then the patient is asked to return to the clinic when withdrawal symptoms appear or at night (about 12 hours later). According to the half-life of methadone (about 12 hours), it can be maintained until the second Daily (about 12h later) methadone administration. On the second day, the first 2 doses can be added together as the dose for each administration, and then depending on the patient’s condition, increase by 5 mg per day until the patient’s withdrawal symptoms can be fully controlled and the patient feels well. |
| 1. Maintenance period and maintenance dose | The maintenance period refers to the period during which the dosage of methadone is relatively stable after the introduction of adjustments for 20 to 30 days. It could be months, years or even whole life. The maintenance dose is usually 60-100mg/day, which varies from person to person. |
| 1. How to take | Oral, the patient must take the medicine in the outpatient clinic before leaving. It is forbidden to take medicine out of the clinic. |
| 1. Time to take | During the maintenance treatment period, patients need to go to the outpatient clinic to take medication every day, and the outpatient medical staff will record the daily medication time and dosage. |
| 1. Reduction and withdrawal | When patients undergo methadone maintenance treatment for a period of time, as their physical condition, occupational ability, family function and social function gradually improve, and gradually stabilize, they can take the initiative to reduce the dose of methadone, and those with mild illness may even be put forward a request for drug withdrawal. |
