# Supplementary material for: Clinical and Economic Evaluation of Acupuncture for Opioid-Dependent Patients Receiving Methadone Maintenance Treatment: The Integrative Clinical Trial and Evidence-Based Data
Source: Front Public Health. 2021 Aug 16;9:689753. doi: 10.3389/fpubh.2021.689753 (PMC8415360; doi:10.3389/fpubh.2021.689753)
Supplement: Supplementary file 3 [file Table_2.docx]

**Appendix 3: Health Utility Value**

**（**$\bar{\mathbf{X}}\boldsymbol{\pm SD}$**）**

| Follow-up |  | Exposed group | |  | Control group | | Difference | | *F* Value |  | *P* Value | |  |
| --- | --- | --- | --- | --- | --- | --- | --- | --- | --- | --- | --- | --- | --- |
| week 0 |  | 0.621±0.100 | |  | 0.627±0.099 | | -0.006±0.019 | | 0.088 |  | 0.768 | |  |
| Week 4 |  | 0.628±0.101 | |  | 0.599±0.099 | | 0.032±0.019 | | 2.782 |  | 0.101 | |  |
| Week 6 |  | 0.640±0.136 | |  | 0.607±0.090 | | 0.027±0.020 | | 1.806 |  | 0.184 | |  |
|  |  | |  | | |  | | Group | 1.489 |  | | 0.227 | |
|  |  | |  | | |  | | Time | 0.385 |  | | 0.681 | |
|  |  | |  | | | Group×Time | | | 1.712 |  | | 0.185 | |

NOTE. The health utility value is calculated based on the British SF-6D scoring model; repeated measures ANOVA, Greenhouse-Geisser correction is performed when the spherical symmetry condition is not met.
